# Preservation and adaptation: a qualitative comparison of Japanese and Dutch kendo instructors

**DOI:** 10.3389/fspor.2025.1688333

**Published:** 2025-11-26

**Authors:** Pepijn Boomgaard, Kohei Kawashima

**Affiliations:** Graduate School of Sport Sciences, Waseda University, Tokyo, Japan

**Keywords:** kendo, martial arts, budo, sports coaching, Japanese culture, Dutch culture, cultural dimensions, qualitative research

## Abstract

**Introduction:**

Kendo, a Japanese martial art rooted in traditional swordsmanship, has spread internationally. The art maintains strong cultural ties to Japan. As the global practitioner base expands, questions arise about how kendo is adapted in different cultural contexts and to what extent its original values and traditions are preserved. This qualitative study explored how cultural background influences kendo instruction by comparing the perspectives of Japanese and Dutch kendo instructors.

**Method:**

Ten experienced kendo instructors from Japan and the Netherlands participated in semi-structured interviews. Data were analyzed using reflexive thematic analysis. Hofstede's cultural dimensions theory served as the analytical framework for interpreting the influence of national culture on instructors’ teaching approaches.

**Results:**

Japanese instructors emphasized competitive success, hierarchical relationships, and student creativity, reflecting cultural values of masculinity and high power distance, while also subverting expectations regarding individualism. In contrast, Dutch instructors promoted recreational practice and egalitarian relationships, and reported challenges with overly critical students. These findings mirror cultural tendencies of femininity and low power distance, but defy assumptions regarding individualism. Despite these differences, both groups showed a strong commitment to preserving kendo's traditional values.

**Discussion:**

The findings suggest that while instructional styles vary by culture, kendo's traditional principles are maintained across borders. Instructors play a key role in negotiating the balance between cultural adaptation and the preservation of kendo's identity. This study contributes to understanding the cross-cultural transmission of martial arts and highlights the need for further research including student perspectives and other national contexts.

## 1 Introduction

Kendo is a Japanese martial art based on traditional swordsmanship, and it is deeply rooted in the cultural values and traditions of Japan. Nowadays, kendo is not just treated as a sport but is also used as a tool to promote human character and values (1). The martial art is not only widely practiced across Japan but has also become increasingly popular globally. Although kendo's governing bodies welcome this global spread, they hope to preserve kendo's unique properties during the process (2). Consequently, global kendo is currently still centered around Japan (3), with Japanese terminology used around the world (4, 5). Japanese instructors often teach at seminars aimed at non-Japanese practitioners and many non-Japanese practitioners visit Japan to learn more about kendo (5, 6). As the number of non-Japanese practitioners increases, it is important to find out how kendo is adapted into different cultures and to what degree its intrinsic values and traditions are maintained, an issue that is currently underexplored.

Kendo has a long history in Japan, and as of 2025, the All Japan Kendo Federation reports approximately two million registered members (about 1.62% of Japan's population) (7). In addition, many Japanese students are introduced to kendo through mandatory martial arts classes in junior high school (1). In Japan, many practitioners begin training at a young age, with youth sport participation often organized through extracurricular school clubs (8). Success in high school competitions can offer students access to university athletic recommendation systems, providing an academic pathway through kendo (9). Beyond education, kendo proficiency can also support career prospects, particularly in professions such as physical education teaching, law enforcement, and correctional services, where continued practice is integrated into professional life (10).

Although the Netherlands has a relatively long history of kendo, with the Dutch Kendo Federation (NKR) established in 1966 (11), popularity remains limited. As of 2024, the NKR reported only 367 registered kendo practitioners (about 0.2% of the Dutch population), of whom just 42 were youth members (12). In the Netherlands, sports are typically practiced within local clubs that operate independently from the formal education system (13). There are no professional instructors or competitors in the Dutch kendo community. Kendo clubs are generally volunteer-run, with the most experienced practitioners assuming instructional roles. Like in most European countries (14), people of all ages and levels practice together. Despite its longstanding presence, kendo remains relatively unknown to the broader public, and competitive success offers little to no tangible benefits in terms of academic or career advancement.

Previous research shows that many non-Japanese kendo practitioners started because they were interested in learning about Japan and Japanese culture through kendo and that they are drawn to the spiritual aspects of the art (15), and that non-Japanese practitioners placed more importance on “spiritual development” and “discipline” in kendo practice than their Japanese peers, who favored “skill improvement” and “interpersonal relationships” (16). European practitioners were found to be interested in learning about spiritual training and self-discipline, the relationship between body and mind, and learning proper traditional techniques through kendo (17). Furthermore, studies have shown that practitioners of martial arts in Western contexts frequently incorporate Japanese terminology into their training routines (18).

There is great importance placed on the role of instructors in kendo, as they are the ones responsible for their students’ perception of kendo and passing on the traditions associated with the art (19). Japanese instructors have been found to successfully pass on the importance of self-improvement, traditional techniques, and the value of Japanese tradition and etiquette in kendo to their students (20). It can be concluded that it is the instructors who are responsible for ensuring that kendo's values are preserved as it spreads across the world. It would be valuable to look at the differences between Japanese and Western kendo instructors.

Culture plays a central role in how personal values and social relationships take shape in a society (21). Culture has been found to affect leadership, attitude towards sport coaches, and motivation in sports (22–25). Although the Netherlands and Japan share similar levels of economic and educational development, the two countries’ national cultures differ sharply in many aspects (26, 27). Comparing these two countries thus offers a valuable opportunity to examine how cultural background affects kendo instruction.

Hofstede's (28) theory of cultural dimensions is used as analytical lens to structure this comparison. This theory identifies six key aspects of culture: power distance, individualism, masculinity, uncertainty avoidance, long-term orientation, and indulgence. Each dimension provides insight into the values and behaviors prevalent within a given society. The dimensions for Japan and the Netherlands as found by Hofstede (26, 28, 29) are as follows:

Power distance refers to the extent to which inequality and hierarchical structures are accepted within a society. Japan can be characterized as moderately hierarchical, displaying respect for authority while maintaining some egalitarian tendencies. In contrast, the Netherlands is generally considered a highly egalitarian society, where power is more evenly distributed and hierarchy is less emphasized.

Individualism captures the degree to which individuals are integrated into groups. Japan leans toward collectivism, emphasizing group cohesion and social harmony, although not as strongly as some other East Asian cultures. The Netherlands, by contrast, is a highly individualistic society, prioritizing personal autonomy and self-expression.

Masculinity measures the value placed on competitiveness, achievement, and success. Japan ranks among the most masculine cultures globally, where success is often linked to group competition and corporate achievement. The Netherlands, however, is considered a feminine culture, where values such as cooperation, equality, and quality of life take precedence over competition and material success.

Uncertainty avoidance reflects the degree of comfort with ambiguity and unpredictability. Japan scores extremely high on this dimension, indicating a strong preference for structure, ritual, and predictability, along with a resistance to change. The Netherlands also exhibits a relatively high level of uncertainty avoidance, though to a lesser extent. This manifests in a preference for planning and a mild intolerance of unconventional behavior.

Long-term orientation assesses a society's focus on future rewards rather than short-term gains. Japan demonstrates a pronounced long-term orientation, which is evident in the cultural emphasis on perseverance, hard work, and organizational loyalty. The Netherlands, while open to adaptation and progressive in addressing contemporary challenges, tends to place less emphasis on long-term institutional goals, with individuals prioritizing present concerns over future-oriented commitments.

Indulgence concerns the extent to which societies allow for the gratification of desires and enjoyment of life. Japanese culture is marked by restraint, where social norms encourage the control of impulses and place less importance on leisure time. In contrast, Dutch culture is highly indulgent, valuing freedom, personal enjoyment, and the pursuit of a balanced and pleasurable lifestyle. Table 1 summarizes the comparison of Hofstede's cultural dimensions between Japan and the Netherlands. By looking at the values of Dutch kendo instructors, insight can be gained on how people of Western backgrounds perceive kendo and its values.

**Table 1:** Hofstede dimensions of Japan and The Netherlands.

Dimension	Japan	Netherlands
Power distance	Moderately hierarchical	Highly equalitarian
Individualism	Moderately collectivist	Highly individualistic
Masculinity	Highly masculine	Highly feminine
Uncertainty avoidance	Highly uncertainty avoidant	Moderately uncertainty avoidant
Long-term orientation	Highly long-term oriented	Moderately long-term oriented
Indulgence	Highly restrained	Highly indulgent

While existing research on kendo has yielded valuable insights, much of it has relied on quantitative methods, which, although informative, may not capture the nuanced experiences that shape the transmission of kendo across cultures. Some qualitative studies have begun to explore the experiences of kendo practitioners, such as Sato's (30) phenomenological study on kendo competitors and Hernández Chávez's (31) (auto)ethnographic study on kendo in Chile and Spain. Additionally, qualitative research has been done on international practitioners in other Japanese martial arts, such as Xie et al's (32) study on Chinese karate practitioners and Dykhuizen's (33) mixed-method cultural comparison of aikido in Japan and the United States. Still, the role of instructors, who play an important role in preserving and passing on traditional practices, remains largely underexamined. Specifically, no studies to date have examined how instructors from different cultural backgrounds approach teaching kendo, nor how cultural norms influence their perceptions of authority, tradition, and pedagogy. As kendo continues to expand globally, understanding how its core values are interpreted and preserved in diverse settings is increasingly important. This study seeks to address this gap by exploring how cultural background influences instructional approaches in kendo, focusing on a comparative analysis of Japanese and Dutch kendo instructors. In doing so, it contributes to a deeper understanding of how traditional martial arts are taught, adapted, and sustained across cultures.

## 2 Method

### 2.1 Research design

This study adopted a qualitative research design with a constructivist paradigm, aiming to explore how individuals make meaning of their experiences within culturally embedded practices. A qualitative approach was chosen to allow for rich, in-depth exploration of participants’ perspectives on kendo instruction. Data was gathered through in-depth interviews. Reflexive thematic analysis (34) was used as the analytical framework, as it is a flexible method for identifying, analyzing, and interpreting patterns of meaning within the responses of the participants. This approach allowed for an in-depth exploration of participants’ experiences, perspectives, and beliefs.

This research was approved by the University of Tsukuba Institutional Review Board, where the first author was enrolled at the time of data collection. Participants were not compensated for participating in the study. All participants provided informed consent prior to participating in the interviews.

### 2.2 Researcher positionality

The first researcher in this study is a white Dutch male who has spent many years living in Japan and has personally experienced kendo practice in both Japan and the Netherlands. As a result, he is familiar with both countries’ cultures and training environments. This influenced his reflections and interpretations during the analysis, and thus continued reflexivity was used to stay grounded in participants’ experiences. The second researcher is a Japanese male, who has previously lived in the United States. Although he has been involved with research on Japanese martial arts before, he is not personally an experienced kendo practitioner. This allowed him to approach the research with an outsider perspective and to analyze the data in an objective manner.

### 2.3 Participants

Participants were recruited through convenience sampling. Care was taken to ensure a diverse participant pool by including individuals from a variety of backgrounds and teaching at different locations. Five Japanese and five Dutch kendo instructors were interviewed. Inclusion criteria were individuals with an instructor rank (6th dan grade or higher) who had more than 10 years of experience teaching kendo. The average age of the participants was 61 (SD = 9.8). Nine of the participants were male, one was female. On average, participants had 29.4 years of teaching experience (SD = 11.5).

Recruitment was conducted through a combination of face-to-face and online inquiries. After interviews with five participants in each group, data saturation was deemed to have been reached, as responses began to converge and similar ideas were consistently expressed across topics. This relatively early point of saturation may be attributed to the homogeneity of the participant group, as all were experienced kendo instructors (35, p. 13–4).

All interviews with Japanese participants were conducted in person at locations of their choosing. Interviews with Dutch participants were conducted online via Zoom. To protect participant confidentiality, pseudonyms were used. Relevant participant characteristics are presented in Table 2.

**Table 2:** Characteristics of participants.

Participant	Age	Gender	Teaching experience	Nationality
Sato	71	Male	49 years	Japanese
Suzuki	52	Male	22 years	Japanese
Takahashi	56	Female	10 years	Japanese
Tanaka	54	Male	28 years	Japanese
Ito	72	Male	32 years	Japanese
Van Dijk	47	Male	23 years	Dutch
Dekker	67	Male	36 years	Dutch
De Vries	53	Male	18 years	Dutch
Mulder	75	Male	36 years	Dutch
De Wit	63	Male	40 years	Dutch

### 2.4 Data collection

Semi-structured interviews were used to collect the data. Interviews with Japanese participants were conducted in Japanese, and interviews with Dutch participants were conducted in Dutch. The average interview duration was 88 min (SD = 33.6). Participants were asked open-ended questions about their experiences as kendo instructors, including their teaching style, their relationships with students, and their overall perspective on kendo. Additionally, as all Japanese instructors had experience teaching kendo abroad, they were asked about these experiences. On the other hand, all Dutch instructors had experienced kendo training in Japan and were asked about these experiences. The in-person interviews were recorded using a voice recorder, and online interviews were recorded using Zoom's built-in recording function. All recordings were transcribed using the Office 365 transcription tool and subsequently reviewed and manually corrected to ensure accuracy.

### 2.5 Data analysis

The data were analyzed by the researchers using reflexive thematic analysis following Braun and Clarke's (34) six-phase approach: (1) dataset familiarization, (2) data coding, (3) initial theme generation, (4) theme development and review, (5) theme refining, defining, and naming, and (6) writing up. First, the researcher listened to the audio recordings and read the transcripts in the original languages. Notes were taken on salient observations in the data. Next, semantic and latent codes were developed based on the notes. Then, the transcripts were coded. Codes were then sorted and grouped based on similarity. After this, initial themes were generated by combining related codes. Themes relevant to both participant groups were selected for further analysis. These final themes were then used to highlight differences and similarities between Japanese and Dutch participants.

### 2.6 Translation

The interviews were conducted in either Dutch or Japanese. The transcripts were analyzed in their original language. Only the quotes used in the final paper were translated into English. Both authors worked on the translation. Care was taken to preserve the original meaning, tone, and context of the statements.

## 3 Results

Three broad themes were created: (1) Meaning of Kendo, in which participants discuss the role kendo plays in their lives, (2) Instructor-Student Dynamics, in which participants describe the relationships they have with their students, and (3) Navigating Student Culture, in which participants reflect on how the culture of their students affects their teaching style. All three themes were then accordingly separated into subthemes.

### 3.1 Theme 1: meaning of kendo

This theme relates to how both groups of participants perceive kendo and the role it plays in their professional and private lives. They also discussed the concept of kendo as a form of budo, a Japanese martial art with traditional values.

#### 3.1.1 Theme 1.1: kendo as a career

Japanese participants discussed the fact that competition in kendo is important, as it affects a practitioner's professional prospects. Even though kendo is often said to be a budo rather than a sport, the competitive side of the art is of utmost importance in Japan. Sato (Japanese) described how competitive kendo, in practice, already functions as a sport, but that this is not necessarily problematic:

I think that for young people, it's fine to treat it as a sport. And as they get older, as their dan grade goes up, it will naturally turn into budo. … So, of course, to some extent, basic etiquette and politeness should be preserved, but as a competition, as a sport. … The All Japan Championship is already a sport, right?

Participants mentioned how competitive success in kendo is important in Japan because it can lead to scholarships at good schools and universities, as well as employment opportunities. High schools and universities across Japan actively recruit top kendo practitioners by offering generous scholarships. Good competitive results can even lead to employment, as prefectural police departments have special divisions where personnel can dedicate themselves fully to kendo practice, and certain companies also have their own kendo clubs. Ito (Japanese) explained why winning in kendo is so important:

High school teachers want their students to win. College students too, they want them to win college competitions. As I said before, if you win a lot of a lot of competitions, you will be able to get a job at a good place as a result.

Tanaka (Japanese) highlighted how this link between kendo competition and career prospects distinguishes kendo in Japan from kendo in other countries:

Among Japanese people, the top athletes are the ones whose lives are changed by winning. I strongly feel that they put their own … lives and future on the line when they fight. When I go abroad … they don't put their life on the line as much. … Basically, even if they don't win in kendo, the majority can still make a living. In that respect, victory or defeat, fighting with their livelihood on the line. I do feel like that difference is there.

In Japan, the outcome of kendo competitions can have a great effect on practitioners’ lives. Major tournaments are approached as serious sporting events, with strong performances opening doors to educational and professional advancement. This illustrates how kendo operates within a broader socio-economic system that rewards competitive success. The accounts shared by participants reveal that for many, especially younger practitioners, kendo is not only a martial art but a pathway toward tangible life opportunities. The emphasis on winning reflects how kendo in Japan has been institutionalized within schools, universities, and workplaces, where performance outcomes can determine access to scholarships, jobs, and social status. This duality highlights how kendo is struggling with its identity as traditional budo.

#### 3.1.2 Theme 1.2: kendo as a hobby

Some Dutch participants seemed to have a similar view of the sports-like competitive side of kendo as the Japanese participants. De Wit (Dutch) explained:

There is respect for your opponent, there is etiquette, and there's no cheering when you score a point, which is allowed in Western sports, but other than that, it's just, you have the rules, and within those rules, you have to try to win. So, in that regard, it's just a sport like volleyball.

However, despite their passion and commitment, Dutch practitioners emphasized that kendo remains a hobby in the Netherlands, rather than a professional or career-oriented pursuit. As a result, Dutch participants see kendo as something that is enjoyed in one's free time. De Vries (Dutch) explained how kendo is widely recognized in the Netherlands:

“Guys, it's your hobby, yeah?” And I don't mean to downplay it, but it's very simple. If you go to your employer on Monday and tell them, “I just became third or fourth dan [grade],” you'll have to explain what it is first.

This distinction significantly shapes their relationship to the practice. Dutch instructors prioritize enjoyment and sustainability in training. They recognize that, because kendo is practiced in practitioners’ free time, it must remain pleasant to retain members. Dekker (Dutch) emphasized this point, stating that even at high levels of practice, the experience should still be fun:

Maybe that is my most important philosophy, that what people do, whatever it is they do, it doesn't matter, but in their free time, because it is their free time, they enjoy doing it. And if you get further and you want to be active in the Dutch [national] team, yes, a bit more might be asked of you, but it should still be pleasant to be a part of it.

De Wit (Dutch) shared a similar view, explaining his approach to balancing intensity with enjoyment: “Yes, my motto is … gei ni asobu [play in art]. … I try to make people practice very hard without them noticing it. But they do need to enjoy it.” This approach is partly a response to the fact that student retention is a big challenge in the Netherlands. Dutch kendo clubs face ongoing difficulties in both attracting new members and retaining existing ones, with high attrition rates significantly undermining practice quality at times. Dekker (Dutch) said, “During your classes, you have to be aware that you also have to motivate students, that you have to keep the class interesting, otherwise they will walk away.” De Wit (Dutch) recounts his own experience with students quitting after implementing a vigorous training regime he had adapted from Japan, “I remember when I came back from Japan. Of course, I just started teaching what I had done in Japan. And half a year later there were no members left.” Van Dijk (Dutch) highlighted the specific challenge of retaining youth practitioners, particularly when training becomes more demanding and painful:

That's just difficult. You can never force anyone. It's all voluntary. And it's damn hard to keep it fun for children the moment they start hitting each other and it starts hurting. Because that is a big hurdle with kids. And then they have to be quite tough to go for it, I think.

We can see that Dutch participants’ perception of kendo as a hobby shapes how they approach their classes. Although they want to push their students, enjoyment is consistently emphasized as the top priority. Instructors are always aware of the risk of students quitting, and pedagogical strategies are oriented toward creating a positive and engaging training environment. This contrasts with the Japanese context, where institutional support and professional pathways can justify more intense and rigorous training.

#### 3.1.3 Theme 1.3: kendo as a tradition

Although kendo occupies different roles in the lives of Japanese and Dutch practitioners, participants from both groups agreed on the importance of maintaining its traditional values and etiquette. Tanaka (Japanese) talked about the significance of passing on the traditional culture of kendo: “As budo, we must preserve the etiquette, the integrated way of thinking. … I especially want to pass on the etiquette strictly, or rather, properly.” Similarly, Ito (Japanese) stated that the transmission of these values is a principal part of teaching: “My teaching philosophy … my ideal is to teach that [traditional culture] correctly while having my own beliefs.” Takahashi (Japanese) talked about kendo's dual nature, distinguishing between its “competitive side” and “traditional side,” adding that “the fact that it has both of these sides is very interesting.” However, she highlighted the danger of neglecting the cultural aspects of kendo:

The competitive aspect is also very important, but in competitions, especially recently, there has been a tendency for it to be sports-like, technically too. I don't completely reject that, that is also a part of it, but attached to that is also the tradition and culture that is the backbone of kendo, and if we cut that out it would not be kendo anymore, so how do we convey that part?

The Dutch participants expressed similar sentiments regarding the importance of tradition and etiquette. Van Dijk (Dutch) said, “I do think it is important that people follow certain etiquette in kendo, that they always properly do the greetings.” Mulder (Dutch) stressed that this emphasis on discipline and respectful behavior is especially important in the Dutch context, where such values might be unfamiliar to beginners: “So greeting, your behavior. Especially the behavior is important with people who start kendo in the Netherlands. … they might not be able to imagine that discipline is kept so tightly.” Dekker (Dutch) framed kendo's emphasis on respect as part of its character-building value: “You always need to respect your opponent or your training partner. … That formative aspect is why I think judo, kendo, and other Japanese martial arts are important, also for the youth.”

In both Japan and the Netherlands, instructors see the preservation and transmission of kendo's traditional elements as essential to their teaching. In kendo, these values are often expressed through formalized greeting and bowing, showing a mutual respect between practitioners. While the competitive side of kendo continuous to evolve, participants from both groups emphasize that it is the traditional aspects that give kendo its distinctive identity. A tension between modernity and tradition within the martial art can be observed, especially in the Japanese environment.

### 3.2 Theme 2: instructor-student dynamics

This theme explores how participants from both groups describe the relationships they have with their students and how they position themselves as instructors within their respective kendo communities. There were clear differences in how the instructor's role is socially and culturally constructed in Japan and the Netherlands.

#### 3.2.1 Theme 2.1: position as professional teacher

In Japan, kendo is a competitive martial art, and there is institutional demand for high-level coaches, particularly within schools and universities. All Japanese participants in this study are currently, or have previously been, professional kendo instructors. For them, teaching kendo is not merely a personal pursuit but a formal aspect of their occupation. This professional status leads to a sense of distance and hierarchy between the instructors and their students. Japanese instructors emphasized that they approach kendo instruction as part of their professional responsibilities. Tanaka (Japanese) explained, “The students are paying money and taking the classes. And I receive a salary from the government. … I am in a position to pass on kendo to these students.” Other Japanese participants also stated that their official position affects their relationships. Takahashi (Japanese) stated, “I always keep my position as teacher in mind,” while Suzuki (Japanese) said, “Thinking about my age and position … The way I do it. … I guess I keep some distance [between me and the students].

Sato (Japanese) also discussed that students enrolling into strong schools and universities often do so with conviction and certain expectations of their instructors: “I think there is also the issue of whether they will follow the teacher or not. … So, when children join [a strong high school], it means they are convinced by [that school's instructor's] teachings. I don't think anyone is joining a [strong] kendo club whimsically without conviction.” He later added that he thinks it is “difficult to teach at a strong school” because of the pressure that comes with it. This highlights how Japanese kendo instructors serve as central figures whose presence influences student's long-term trajectories.

These comments reveal that Japanese instructors are highly conscious of their professional role as educators or coaches. These positions, often embedded within public institutions such as schools, universities, and law enforcement, come with expectations around authority and responsibility. This structured hierarchy often leads to a degree of distance between them and their students, illustrating that instructor-student dynamics are not only shaped by personal teaching philosophies, but also by their environment.

#### 3.2.2 Theme 2.2: friendship with students

In contrast to the professional and hierarchical relationships described by Japanese participants, Dutch instructors framed their relationships with students in more informal, egalitarian terms. Several Dutch participants characterized their connection with students as being rooted in friendship. Mulder (Dutch) describes the relationship he has with his students as “almost based on friendship.” Similarly, De Wit (Dutch) explained:

Yeah, we just have a very amicable relationship. As for my older students, I’ve been teaching them for 20 or more years now, you know. So, then you also get a private relationship, not just a teacher-student relationship. … So, you go visit each other at home once in a while.

This familiarity was also reflected in how instructors conceptualized their role in the dojo. Rather than seeing themselves as formal teachers, some Dutch instructors described their role as closer to that of a facilitator or training partner. For example, De Vries (Dutch) said that even though he is the one teaching, he does not see himself as a teacher. “I don't have students. … They're all grown-ups or older than me, so they're not students. That whole relationship doesn't exist.”

These statements reveal a markedly different approach to teaching. Dutch instructors emphasized a horizontal dynamic in which mutual respect and shared passion for kendo guide their interactions. While instructors still take responsibility for teaching and guiding practice, the relationships they cultivate with students often blur the lines between teacher, peer, and friend. This clearly contrasts with Japan's formal institutional teaching positions.

#### 3.2.3 Theme 2.3: don't call me sensei

Although Dutch participants expressed a strong appreciation for the traditional aspects of kendo, they did not feel the need to replicate Japanese cultural practices in their entirety. Specifically, they rejected hierarchical forms of address and interaction that are commonly observed in Japan. They emphasized that while they may take on the role of instructor during training sessions, they prefer to be seen as equals outside the dojo. Dekker (Dutch) explained: “In the dojo, okay, I am the one who is in charge, and I hope they listen to me and do the things [I tell them to do], but after that it's finished. Then I'm just the regular person again.” While the instructors themselves try to avoid such behavior, they occasionally observe Dutch practitioners attempting to adopt Japanese customs such as addressing instructor with the honorific sensei. Participants mentioned discoursing this kind of behavior. Van Dijk (Dutch) talked about steering students away from it:

I do always try to take that away a bit, because as you know, I'm not very hierarchical. I don't care for it much. But you do notice that some beginners feel a great need for that. To continuously say sensei this and sensei that. And then I'm like, just call me [by my first name].

De Vries (Dutch) echoed this sentiment more strongly: “I usually give them a middle finger with a big smile when someone starts calling me sensei. I think that's crazy. It doesn't make any sense here in Europe, especially in the Netherlands. Calm down.”

While traditional values in kendo are respected by Dutch instructors, they aim to integrate them into a local context that emphasizes equality, informality, and authenticity. There is a conscious effort to avoid performative mimicry of Japanese culture and to create a training environment suitable for Dutch practitioners, illustrating the careful balance Dutch instructors try to strike between maintaining kendo's traditions and adapting its practice to fit their local values.

### 3.3 Theme 3: navigating student culture

A major theme that emerged among participants from both groups was the importance of recognizing and dealing with the cultural background of students. Instructors must always pay attention to the inherent cultural tendencies of the practitioners they are teaching, as this can influence how their individuality should be managed.

#### 3.3.1 Theme 3.1: encouraging individuality

Japanese participants emphasized the importance of fostering students’ individuality and creativity in kendo. Rather than imposing a fixed style or strictly controlling how techniques are executed, instructors described an approach that encourages self-discovery and personal expression within the framework of traditional kendo. Sato (Japanese) clearly explained: “I don't impose my own kendo style on them at all.” Instead of prescribing a singular way of practicing, Japanese instructors trust their students to learn through observation, experimentation, and self-motivation. Takahashi (Japanese) talked about the fact that many advanced practitioners tend to have a clear idea of what they want to do and are highly autonomous in their development:

The students have their own strong sense of kendo, so even if I don't tell them anything, they will think about and devise various things on their own, and they will try to steal techniques from those around them. So, on the contrary, even if the instructors don't say anything, they will do it by themselves.

Tanaka (Japanese) emphasized the importance of balancing respect for tradition with the freedom to innovate:

If you say, “This is no good, this is no good,” they will lose the ability to come up with new things, right? It's also in the hands of the instructor, isn't it? It's about ingenuity. And using individuality … So, I want to increase their ingenuity and also preserve the tradition, so I always keep in mind that I want to create a form that is beautiful to Japanese kendoka.

These statements reflect a nuanced teaching philosophy in which individuality and creativity are not seen as being at odds with tradition, but as something that can emerge and thrive within it. Japanese instructors appear to cultivate a learning environment where students are encouraged to observe, reflect, and adapt techniques based on their own understanding, rather than passively receiving inflexible instruction.

#### 3.3.2 Theme 3.2: critical students

A common issue raised by Dutch participants was the tendency of Dutch students to be highly critical and questioning toward their instructors. Participants discussed how authority is often met with skepticism, and students are not inclined to follow instructions without first understanding or challenging the reasoning behind them. While this critical thinking can be a strength, instructors sometimes experience it as a barrier to effective teaching and learning. De Vries (Dutch), reflected on how difficult it was to gain acceptance for his ideas, even when he held a formal leadership role:

There were quite a few moments where I thought, “Wait a minute, I want this.” But I know that something won't just happen because I want it to. I can't force it, they're Dutch, right? They always have their opinions. And that's quite an issue and some of those things were never, I never got them to understand those things.

Similarly, Mulder (Dutch) described feeling frustrated when students demand explanations or question instructions instead of simply practicing:

It sometimes is difficult to convince people … You do need to be understanding to do it and sometimes that is difficult. And sometimes, because that is quite a reaction from people, quite irritating. It makes you think, “Ask? Why are you asking this? Why? Don't ask so many questions. Do it. Just do what is being said. … That is something you sometimes run into as a teacher.”

Van Dijk (Dutch) also noted that while critical thinking is important, it can become excessive, undermining the trust that is sometimes necessary in the teacher–student relationship:

You should never just take anything without thinking about it critically. I do think that in the Netherlands we have made a sport out of criticizing everything. While in some cases you just have to think, ‘Wait, he has been with the team for such a long time, he has [done] this, this, and this, and maybe he has a point.

Although Dutch instructors appreciated their students’ intellectual engagement and independence, they also expressed that such attitudes could limit the students’ growth. When students reject guidance or demand justification, the flow of instruction might be disrupted, and the learning process might be hindered.

## 4 Discussion

The analysis revealed three overarching themes that reflect contrasting instructor perspectives shaped by cultural context: (1) Meaning of Kendo, (2) Instructor–Student Dynamics, and (3) Navigating Student Culture. Within these themes, both similarities and differences emerged between Dutch and Japanese participants, which can be meaningfully interpreted through Hofstede's (28) cultural dimensions.

### 4.1 Meaning of kendo

Japanese participants emphasized the institutional role kendo has within Japanese educational and professional environments. Competitive success in major kendo tournaments is often linked to educational and professional opportunities, resulting in a strong emphasis on winning, especially among younger practitioners. Dutch participants, on the other hand, described kendo as a leisure activity practiced in one's free time. They are more concerned with enjoyment and maintaining a welcoming environment to support member retention. These findings are consistent with Iwamoto et al.'s (16) research, which found that Japanese kendo practitioners tend to be more achievement-oriented than their non-Japanese counterparts. They also align with Alfermann et al.'s (22) findings that Japanese swimmers are more strongly motivated by competitive success compared to their German peers.

While part of this difference can be attributed to the relative popularity of kendo in each country, Hofstede's cultural dimensions (28, 29) offer a deeper explanation. Japan scores very high on masculinity, reflecting a culture that values achievement and competition, which is consistent with the emphasis on success among Japanese instructors. In contrast, the Netherlands scores much lower on masculinity, showing a feminine culture emphasizing quality of life, well-being, and work-life balance.

This distinction is also reflected in the indulgence dimension. Japan has a restrained culture, in which discipline and order is valued. The Netherlands has an indulgence culture, valuing leisure and enjoyment. These cultural orientations help explain why Japanese instructors accepted the competitive expectations as part of their responsibilities, while Dutch participants were more concerned with maintaining a healthy atmosphere in which kendo can be enjoyed. Both groups, however, emphasized the importance of tradition and etiquette in kendo. This value is likely rooted in their shared tendency for uncertainty avoidance, which favors predictability and structure. While Dutch instructors may reject hierarchical customs such as the use of the honorific sensei, they still appreciate the predictability and ritual inherent in kendo's practice. Similarly, Japanese instructors emphasized preserving traditional forms while encouraging sports-like competition within kendo's traditional framework.

Interestingly, while previous research has indicated that non-Japanese practitioners of kendo are often drawn to the mental or spiritual dimensions of the martial art (15–17), such themes were notably absent from the interviews in this study. None of the Dutch participants emphasized philosophical or spiritual growth as a core reason for their involvement in kendo, instead mentioning topics such as enjoyment and social connection. However, results of this study do align with findings of those papers regarding Western kendo practitioners regard for kendo's etiquette and manners, as Dutch participants clearly stated the importance of maintaining these facets of kendo.

### 4.2 Instructor-student dynamics

Japanese participants discussed their formal teaching positions, which created a hierarchical distance between them and their students. Interactions are shaped by their position as teacher, and students have expectations regarding their behavior. In Japan, teachers are addressed with formal honorifics, and authority is generally respected. On the other hand, Dutch instructors framed their relationships as equal and amicable, emphasizing mutual respect and informality.

This difference clearly reflects differences in Hofstede's power distance index (28, 29). Japan scores moderately high, aligning with the formal hierarchy observed in participants’ instructor-student relationships. Teachers are respected as authority figures, which is reinforced by their positional status. The Netherlands scores low on power distance, reflecting the egalitarian relationships described by Dutch participants. Dutch instructors tend to downplay their authority and reject the use of overly formal honorifics. These findings align with Alfermann et al.'s (22) observation that Japanese coaches often place limited emphasis on personal relationships with their athletes. They also support Hernández Chávez's (31) finding that hierarchical structures in kendo tend to be less rigid in societies where such systems are not culturally reinforced.

Although previous studies found that a general interest in Japanese culture is one of the most common reasons for Western kendo practitioners to start kendo (15), it seems that participants in this study do not appreciate “overly Japanese” behavior, such as the use of the term sensei, among their students. Some even explicitly rejected attempts by students to behave in what they viewed as inauthentic or exaggerated ways, suggesting a desire to integrate kendo into their own cultural context rather than simply replicate Japanese norms.

### 4.3 Navigating student culture

Both participant groups valued student individuality but approached it differently. Japanese instructors aimed to foster students’ autonomy and resourcefulness by not interfering too much, allowing students to discover techniques and grow on their own. They encouraged students to cultivate creativity within tradition. Dutch instructors, however, often struggled with their students’ critical and questioning attitudes. While they appreciated critical thinking, they felt that excessive questioning could slow down practice and hinder growth.

Within the Hofstede model (28, 29), the Netherlands is one of the most individualistic countries. This, in combination with a low power distance, explains why Dutch students tend to prioritize their own growth and form independent judgments, and are not afraid to voice their opinions. In more extreme cases, this individualism may even lead to indifference towards others (26). Japanese society, on the other hand, is generally collectivist, with a somewhat high power distance. Japanese people “always have to take full responsibility for their behaviour towards others” (26), possibly discouraging individuals from speaking up or challenging authority. As a result, Japanese instructors may feel the need to deliberately encourage individualism in an intentional act to counterbalance social conformity. These findings are interesting, as they show that kendo instructors actively consider and adapt to the cultural norms of their students when teaching, a pattern also found by Hernández Chávez (31) in his research on Chilean and Spanish kendo practitioners. Additionally, this supports Hofstede and Soeters's assertion that (26), “in cultural matters, advantages are also disadvantages.”

In summary, the findings of this study reveal that although Dutch and Japanese instructors share a commitment to the traditional values of kendo, their teaching practices are shaped by distinct cultural norms. Through Hofstede's framework (28, 29), we see how values such as power distance, individualism, masculinity, indulgence, and uncertainty avoidance shape perceptions of authority, competition, and individuality within kendo. Dutch instructors value equality and recreation, while Japanese instructors must concern themselves with hierarchy and competitive achievement. However, both groups ultimately aim to preserve kendo's traditional values while navigating their respective cultural contexts. These findings highlight how universal practices like martial arts are locally adapted and show the value of cultural frameworks in interpreting cross-cultural differences in sports practice and pedagogy.

### 4.4 Implications

This study offers several implications for the international practice and instruction of kendo and other traditional martial arts, particularly in multicultural or cross-cultural teaching environments.

Findings highlight the importance of adapting instructional approaches to align with local cultural values. Japanese instructors teaching abroad, or non-Japanese instructors teaching Japanese martial arts, should be mindful of how cultural dimensions such as power distance, individualism, and masculinity shape expectations around hierarchy, authority, and competitiveness. Being aware of such differences can improve instructor-student dynamics and enhance engagement. As kendo continues to globalize, it might be necessary for kendo to evolve in ways to better suit different cultural contexts. Rather than universally adhering to Japanese norms, instructors should consider which traditional elements are essential to preserve and which may be adapted to fit local customs without losing the art's core identity. In addition to this, it might be vital for Japanese and international governing bodies to clearly define kendo's most important intrinsic values and how they should be maintained throughout this process of internationalization.

Previous research suggests that European practitioners are drawn to the spiritual and cultural aspects of kendo. However, this study's Dutch participants scarcely mentioned such aspects and were critical of students overly mimicking Japanese behavior. Although these studies did not specifically look at Dutch practitioners, this might present a gap between practitioners’ motivations and instructors’ views. It might be worthwhile for Western instructors to also guide students in understanding the history, tradition, and philosophy of kendo, and to explain the meaning and purpose behind kendo's etiquette, rather than just focusing on technical aspects.

### 4.5 Limitations

While this study provides valuable insights into how cultural context shapes kendo instruction, several limitations should be acknowledged. First, the number of participants in both cultural groups was relatively small, consisting predominantly of high-ranking, older individuals. As a result, the findings may not be fully generalizable to the broader population of kendo instructors in either Japan or the Netherlands. For example, younger Japanese instructors might perceive a smaller social or hierarchical divide between them and their students due to closer age proximity and changing generational values. Furthermore, many Japanese participants in this study held formal teaching positions within educational or institutional settings. Instructors teaching in more informal or community-based environments might have different perspectives regarding their authority and responsibilities. It should also be acknowledged that the number of female participants in this study was limited. Future research would benefit from including a larger and more balanced sample to better capture gender-related perspectives.

Additionally, this study focused exclusively on instructor viewpoints. While their perspectives are crucial, the lack of student voices means that the perceptions general practitioners have regarding matters such as instructor-student relations cannot be assessed. Incorporating student perspectives could provide a more holistic understanding of the instructor-student relationship, especially in cross-cultural contexts.

Also, this study did not fully address why many traditional practices and cultural norms of Japanese kendo appear to persist even when transplanted into different cultural environments. Despite the clear contrasts in social norms and sporting cultures, the fundamental etiquette, structure, and pedagogical approaches of kendo seem to be largely maintained.

Future studies could broaden their scope by including a more diverse and extensive range of countries, particularly those outside the traditional centers of kendo practice. This would give insight into how kendo is interpreted, adapted, and integrated within a variety of cultural, social, and institutional contexts. This could reveal unique adaptations or tensions between traditional Japanese values and local cultural norms, expanding understanding of how kendo functions globally as both a martial art and a vehicle for cultural transmission. Additionally, incorporating quantitative research could provide valuable complementary insights by measuring attitudes and values of coaches and students on a larger scale and investigate the mechanisms behind the cultural resilience of kendo's traditional aspects.

## 5 Conclusion

This study explored cultural differences between Dutch and Japanese kendo instructors, a topic unexplored yet. Through reflexive thematic analysis of ten in-depth interviews with experienced Japanese and Dutch kendo instructors, three key themes emerged: (1) Meaning of Kendo, (2) Instructor-Student Dynamics, and (3) Navigating Student Culture. Japanese instructors are always conscious of their institutional status and the responsibilities and expectations that come with their position. They must guide their students toward competitive success and tend to maintain a certain hierarchy within the dojo. In contrast, Dutch instructors viewed kendo as a leisure activity, prioritizing enjoyment, student retention, and egalitarian relationships with students. When analyzed through Hofstede's cultural dimensions, these contrasts seem to reflect broader cultural differences in values such as masculinity, indulgence, power distance, and individualism. Despite these differences, both Japanese and Dutch participants stressed the importance of kendo's tradition and culture, although Dutch instructors rejected “overly Japanese” behavior among students. Overall, the findings highlight the complex interplay between the intrinsic values of Japanese martial arts and global cultural frameworks. As kendo continues to develop globally, a deeper awareness of these cultural dynamics can help instructors adapt more effectively to diverse practitioner populations while maintaining kendo's fundamental principles.

## Data Availability

The datasets presented in this article are not readily available because of privacy concerns. Requests to access the datasets should be directed to pepijn.boomgaard@asagi.waseda.jp.
